# Structure, Function, and Therapeutic Use of IgM Antibodies

**DOI:** 10.3390/antib9040053

**Published:** 2020-10-13

**Authors:** Bruce A. Keyt, Ramesh Baliga, Angus M. Sinclair, Stephen F. Carroll, Marvin S. Peterson

**Affiliations:** IGM Biosciences Inc, 325 East Middlefield Road, Mountain View, CA 94043, USA; ramesh@igmbio.com (R.B.); asinclair@igmbio.com (A.M.S.); steve@igmbio.com (S.F.C.); mpeterson@igmbio.com (M.S.P.)

**Keywords:** IgM (immunoglobulin M), hexameric, pentameric, polymeric, polyvalency, joining chain (J-chain), avidity, complement dependent cytotoxicity (CDC), poly Ig receptor (pIgR)

## Abstract

Natural immunoglobulin M (IgM) antibodies are pentameric or hexameric macro-immunoglobulins and have been highly conserved during evolution. IgMs are initially expressed during B cell ontogeny and are the first antibodies secreted following exposure to foreign antigens. The IgM multimer has either 10 (pentamer) or 12 (hexamer) antigen binding domains consisting of paired µ heavy chains with four constant domains, each with a single variable domain, paired with a corresponding light chain. Although the antigen binding affinities of natural IgM antibodies are typically lower than IgG, their polyvalency allows for high avidity binding and efficient engagement of complement to induce complement-dependent cell lysis. The high avidity of IgM antibodies renders them particularly efficient at binding antigens present at low levels, and non-protein antigens, for example, carbohydrates or lipids present on microbial surfaces. Pentameric IgM antibodies also contain a joining (J) chain that stabilizes the pentameric structure and enables binding to several receptors. One such receptor, the polymeric immunoglobulin receptor (pIgR), is responsible for transcytosis from the vasculature to the mucosal surfaces of the lung and gastrointestinal tract. Several naturally occurring IgM antibodies have been explored as therapeutics in clinical trials, and a new class of molecules, engineered IgM antibodies with enhanced binding and/or additional functional properties are being evaluated in humans. Here, we review the considerable progress that has been made regarding the understanding of biology, structure, function, manufacturing, and therapeutic potential of IgM antibodies since their discovery more than 80 years ago.

## 1. Introduction to Immunoglobulin M (IgM)

**Figure 1:**
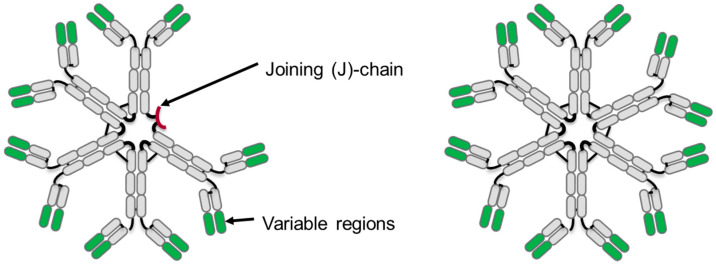
Schematic diagram of an immunoglobulin M (IgM) antibody pentamer (**left**) and hexamer (**right**). Constant regions are shown in gray and variable regions in green, and also shown on the IgM pentamer is the small joining chain (J-chain) in red.

During humoral immune responses, immunoglobulins of the IgM, IgD, IgG, IgA, and IgE isotypes may be produced, each expressing a unique profile of effector functions capable of mediating host defense against invading pathogens. Macro-immunoglobulin, IgM, is initially produced as a surface bound molecule and is expressed in early B cell differentiation. Later in the immune response, IgM is produced by plasma cells and secreted as soluble pentamers that contain 10 antigen binding sites and the joining (J) chain, or as hexamers containing 12 antigen binding sites and no joining chain (J-chain). IgM has a molecular weight of approximately 900 or 1050 kDa for the pentamer or hexamer, respectively (Figure 1).

Due to the polyvalent nature of IgMs, they may exhibit higher avidity for antigen than the bivalent IgG. In addition to neutralizing pathogens, IgM antibodies are highly effective at engaging complement to target lysis of cells and pathogens.

Our understanding of the biology, structure, and function relationships for IgM antibodies has progressed to the point where this antibody isotype can be exploited therapeutically; however, challenges associated with their manufacture remain. Here, we review the progress and the therapeutic potential for this class of antibodies, as well as the potential for new classes of engineered IgM antibodies.

### 1.1. History and Discovery of IgM

Humoral immunity has been studied since the late 1800s when George Nuttall [1] discovered that animal immune sera could kill bacteria. Subsequent analysis of the immune serum using technologies such as electrophoresis and ultracentrifugation allowed for biochemical characterization of the various proteins that could mediate immunity, resulting in the discovery of immunoglobulins. Originally, these serum components were assigned as α-globulin, β-globulin, and γ-globulin fractions to designate the proteins by order of electrophoretic mobility [2]. The first description of IgM antibodies was reported in 1939 by Kabat et al. [3] who evaluated the molecular weight of antibodies produced in horse, cow, pig, monkey, and human serum after immunization with pneumococcus. Due to the large size (approximately 990 kDa), the new antibody was referred to as γ-macroglobulin. In 1944, γ-macroglobulins were also discovered to be expressed at high levels in multiple myeloma patients by Waldenstrom and later independently by Kunkel [4,5]. They identified that the γ-macroglobulin in patient sera migrated close to β-globulin using immuno-electrophoresis and ultracentrifugation techniques. In the 1960s, methods were developed to induce plasmacytomas in mice that produced uniform immunoglobulins that included γ-macroglobulin producing plasmacytomas, recapitulating the data observed in multiple myeloma patients [6]. As the immunoglobulins discovered during this time were being given arbitrary names, in 1964 the World Health Organization defined a nomenclature system for antibody isotypes. As a consequence γ-macroglobulin was renamed IgM, and the M referred to “macroglobulin” [7].

### 1.2. Evolution of IgM Antibodies

**Figure 2:**
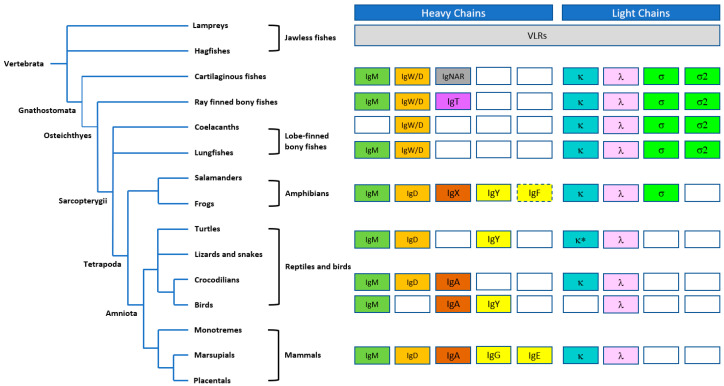
Schematic diagram illustrating the evolution of immunoglobin (Ig) heavy and light chain isotypes in vertebrates, with the IgM isotype broadly represented across phyla. Antigen-binding variable lymphocyte receptors (VLRs) in jawless fishes (agnathans) are thought to be precursors of immunoglobulins. The IgW isotype in cartilaginous fishes is orthologous to IgD in other groups; IgNAR is a “new antigen receptor” isotype, identified in nurse shark, that does not associate with light chains and does not have an ortholog in higher species. IgT appears to be the most ancient Ig specialized for mucosal protection. IgX, originally identified in *Xenopus* is orthologous and functionally analogous to IgA. IgY is the amphibian, reptilian, and avian equivalent of IgG and IgE. IgF only has two constant domains but has homology to IgY. Open boxes represent the lack of certain heavy or light chains in the certain vertebrate lineages; dashed boxes represent a common ancestry; and * represents the lack of κ light chain in snakes. Figure adapted from Pettinello and Dooley 2014 [22] and Kaetzel 2014 [23].

Immunoglobulins, including IgM antibodies, are found in all jawed vertebrates (gnathosomes) that diverged in evolution from jawless fish (agnathans) approximately 550 million years ago [8,9]. Similar to mammals, IgM expression precedes the expression of other antibody isotypes, although, in teleost fish, IgD and IgT are the only other isotypes present [10]. The phylogeny of the immunoglobulin heavy and light chain isotypes is illustrated in Figure 2. However, within certain species, there are distinct differences in the structure of the IgM antibodies produced [11]. For example, the predominant form of IgM antibodies in mice and humans are pentameric in structure and include a J-chain that stabilizes the pentamer, but hexamers and monomers can also be detected [12,13]. However, IgM antibodies in frogs, for example, *Xenopus,* are hexameric in structure even though *Xenopus* IgM has been reported to contain a J-chain [14]. In contrast, IgM from bony fish predominantly forms a tetramer structure, whereas the IgM produced by cartilaginous fish, such as shark, are pentameric in structure [15,16]. It is unclear why hexamer IgM was produced, but it was possible that J-chain synthesis could be limiting [17]. In addition, there are examples of IgM where the expressed µ chain lacks the cysteine in the tailpiece required for proper insertion into the IgM structure [18,19]. Interestingly, in humans and mice, pentameric IgM may also be present that does not contain a J-chain [20]. In fact, we have observed hexamer, i.e., pentamer mixtures produced from recombinant IgM derived from CHO cells, in the absence of transfected J-chain ([21] and unpublished observations).

### 1.3. Ontogeny of B Cells and IgM Antibodies

In mammals, B cell development occurs in a hierarchical, ordered manner in fetal liver during embryonic development, and then in bone marrow and peripheral lymphoid tissue in adults. Within bone marrow, CD34^+^ multipotent progenitors differentiate into common lymphoid precursors (CLP) that give rise to both B and T cell lineages. CLPs subsequently differentiate into early pro-B cells that express Igα and Igβ, essential signaling components of the B cell receptor (BCR). Initiation of the µ heavy chain (µHC) locus rearrangement occurs during the transition to pro-B cells when the RAG1/2 recombination complex induces rearrangement of the D to J_H_ gene segments and, subsequently, to the V to DJ_H_ gene segments. However, no surface µHC is expressed until the cells differentiate into large pre-B cells that express a pre-BCR composed of a µHC complexed to surrogate light chains VpreB and λ5 chain. Signaling through the pre-BCR results in the proliferation, differentiation, and subsequent surrogate light chain downregulation, paving the way for λ or κ light chain rearrangement to form surface IgM with different antigen specificities [24]. Receptor editing and selection occurs at this point and surface IgM expressing immature B cells egress from the bone marrow into the spleen.

Within the spleen, IgM expressing immature B cells begin to express surface IgD, which separate into the following different populations: IgM^low^ IgD^high^ in the follicles and IgM^high^ IgD^low^ in the marginal zone of the spleen. B-1 cells also mature into IgM^high^ IgD^low^ cells. Although the variable domains of the IgM and IgD are identical, alternative transcription and splicing results in both IgM and IgD heavy chains [25]. These populations of naïve B cells are now mature and are poised for clonal expansion and somatic hypermutation upon an encounter with antigen. Upon antigen binding, signaling is initiated through the IgM and/or IgD BCR, resulting in a signaling cascade involving Lyn, Syk, Src, Btk, PLCγ2, and PI3Kδ via co-receptor CD19, resulting in activation, proliferation, and differentiation of B cells that produce secreted IgM, IgG, IgA, or IgE [26,27,28,29]. However, B-1 cells only produce secreted IgM and are described below (see Section 2.1, innate immunity).

During human fetal development, IgM can be detected in the serum at approximately 20 weeks of gestation [30]. In contrast to IgGs, IgM antibodies are not transported across the placenta [31,32]. These fetal IgM antibodies are predominantly polyreactive “natural” IgM antibodies that play a role in the innate defense against infectious pathogens [33]. Postnatal IgM concentrations increase rapidly within the first month of postnatal life likely due to increased exposure to foreign antigens, and then gradually level off [34]. Levels of prenatal IgM are approximately 5 mg/dL in infants at 28 weeks and 11 mg/dL at birth. At one year of age, concentrations of IgM in infants are approximately 60% of that in an adult which is approximately 140 mg/dL, representing approximately 10% of total plasma immunoglobulins [35,36].

## 2. Biology of IgM

### 2.1. Innate Immunity

“Natural IgM” antibodies represent the majority of secreted IgM antibodies found in normal serum and are also located in the pleural and peritoneal compartments [37,38]. This class of IgM antibodies are evolutionarily conserved in all jawed vertebrates [10], are spontaneously produced by a subset of B cells, and often bind to specific antigens in the absence of immunization [39]. Natural IgMs are encoded by unmutated germline variable gene segments with polyreactive binding specificities to epitopes that are generally self- and non-self-antigens [10]. As previously described, these polyreactive IgMs are found at higher frequencies in neonates than adults, both in humans and mice [40].

The source of natural IgM antibodies is somewhat controversial. In mice, natural IgM antibodies are reported to be produced by B-1 cells residing in the bone marrow and spleen [41,42,43,44,45]. However, others have reported that non-B1 plasma cells in bone marrow were the source of murine natural IgM antibodies [46]. In humans, the B-1 cell population has not been studied as thoroughly as the murine B-1 population, but the human B-1 cells are also believed to be the source of natural IgMs [47].

One of the roles of natural IgM antibodies includes the targeting of altered self-antigens or neo-epitopes on dying cells for targeted removal, thereby maintaining tissue homeostasis [48]. One such antigen recognized by natural IgMs to facilitate the removal of apoptotic or dying cells is phosphorylcholine, which is also present on the cell wall of many parasites and microbes [49,50,51,52], thus, providing a first line of defense against pathogens. Carbohydrates, phospholipids, lipopolysaccharide, low-density lipoprotein, plus single and double stranded DNA are other antigen specificities known to be recognized by natural IgM antibodies [48,53,54].

In addition, natural IgM antibodies have been demonstrated to play a role in controlling B cell development, selection, and induction of central tolerance to prevent autoimmunity. The rare condition of selective IgM deficiency in humans, although associated with recurrent infections, is characterized by an increased risk of developing autoimmune diseases such as arthritis and systemic lupus erythematosus [55]. In a study performed by Nguyen et al. [56], secretory µ chain deficient mice (µs−/−) were found to recapitulate the selective IgM deficiency phenotype seen in humans. Although the phenotype may have been due to the reduction in auto-antigen clearance, these knockout mice displayed a block in the differentiation at the pre/pro-B cell stage of development and an escape from central tolerance induction resulting in the accumulation of autoantibody-secreting cells, phenotypes reversible with the administration of polyclonal IgM [56]. Therefore, these data support that natural secreted IgM antibodies facilitate normal B cell development that enforces the negative selection of autoreactive B cells, although the precise mechanism is unclear.

### 2.2. Early Adaptive Immunity

Upon binding to antigen, the BCR expressed on naïve follicular B cells is activated and B cells exit the follicle, proliferate, and produce relatively short-lived IgM secreting plasmoblasts in lymphoid tissues [57]. In the case that activated B cells engage with CD4+ T follicular helper cells through antigen-specific MHC–TCR interactions (major histocompatibility complex and T cell receptor), the B cells will re-enter the follicles, proliferate, and form germinal centers. During this time, the V regions of the BCR undergo somatic hypermutation to “fine tune” the affinity of the antibodies to the specific antigen, and then the antibody heavy chains undergo class switching recombination events to form a variety of isoforms, including IgG1, IgG2, IgG3, IgG4, IgA, or IgE. During this time, the B cells undergo clonal expansion within the follicles, leave the germinal centers, and differentiate into antigen-specific class switched high affinity antibodies producing plasma cells and memory B cells [58].

However, IgM production is not limited to just the initial antigen response. In fact, IgM-expressing memory B cells have been identified that have V region mutations suggesting that these are post germinal center B cells [59,60]. Analysis of peripheral blood has identified that 10 to 20% of all B cells are mutated IgM expressing cells and the IgM expression levels are higher than other less mature IgM expressing B cells [61]. Interestingly, it has recently been described that long-lived murine plasmodium-specific memory B cells include somatically hypermutated IgM expressing B cells [62]. The investigators identified that upon plasmodium rechallenge, the high affinity, somatically hypermutated plasmodium specific IgM+ memory B cells proliferated and gave rise to antibody secreting cells that dominated the early antibody response, via both T cell dependent or independent mechanisms [62].

The interaction of IgM antibodies with antigens can dramatically enhance humoral immune responses to the antigens beyond that of IgG. This is exemplified by studies that have evaluated the co-administration of IgG or IgM with xenogeneic erythrocytes in murine models [63]. When IgGs were co-administered in vivo to mice with xenogeneic erythrocytes, this resulted in suppression of erythrocyte-specific antibody responses [64]. Indeed, this immunosuppressive approach is used clinically to prevent Rh-negative mothers from becoming immunized against Rh-positive fetal erythrocytes, decreasing the incidence of hemolytic disease in newborns. The immunosuppressive mechanism is hypothesized to be epitope masking [64] and does not require complement or IgG Fc receptors [65]. This contrasts with the response observed when IgM antibodies targeting erythrocytes were co-injected in vivo, which resulted in a stronger antibody response against the erythrocytes than when erythrocytes were administered alone [66,67]. Studies have suggested that the activation of complements by the IgM was an important step in this effect, since mice with inactivated complement receptor 1 and 2 have a dampened immune response. For example, when sheep erythrocytes were co-injected with IgM in mice that had the C1q and C3 genes knocked out, antibody responses were not observed or were significantly muted [66]. In addition, similar studies in mice that had complement receptors 1 and 2 (CR1/2) inactivated had a severely impaired antibody response [68]. These data demonstrate that activation of a complement is crucial for the ability of IgM antibodies to feedback enhance antibody responses. IgM antibodies may also increase the concentrations of antigen on follicular dendritic cells in splenic follicles, thereby enhancing antigen presentation and downstream immune response.

IgM antibodies also play key roles in mucosal defense. Secondary lymphoid tissues containing B and T cells, referred to as mucosa-associated lymphoid tissues (MALT), are associated with multiple organ systems including the gastrointestinal and respiratory tracts. These secondary tissues are less organized than primary lymphoid tissues. As discussed below in further detail, the J-chain of pentameric IgM antibodies interacts with the polymeric immunoglobulin receptor (pIgR) on cells which results in the transcytosis of the antibodies from the circulation and through epithelial cells to mucosal surfaces to provide a first line defense against pathogens [69]. The J-chain is also incorporated into the dimeric form of IgA, and it allows efficient mucosal transport [70].

## 3. IgM Antibody Structure

### 3.1. Primary Structure

**Figure 3:**
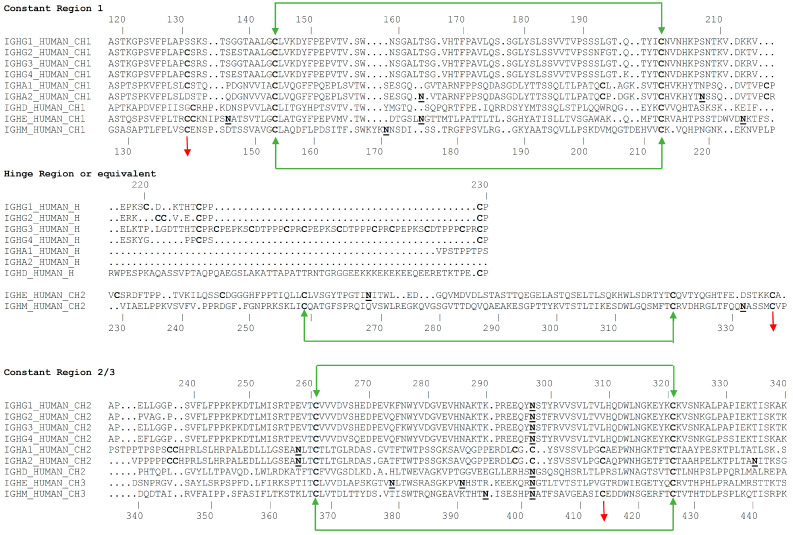

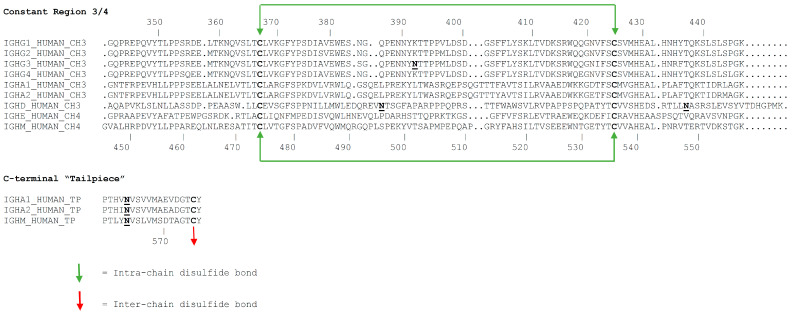
Sequence alignment of the heavy chains of different isotypes of immunoglobulin showing the glycosylation sites and location of inter- and intra-disulfide linkages. The Cμ2 region of IgMs and IgEs is analogous to the hinge regions of the other isotypes. The alignments also highlight, in bold font, the locations of glycosylation sites on each heavy chain. Sequences are numbered according the convention established by Kabat [74].

Antibodies of the IgM isotype are typically found as pentameric or hexameric in format, where each monomer is approximately 190 kDa, comprised of a heavy µ chain with five domains (Vµ, Cµ1, Cµ2, Cµ3, and Cµ4) and a light chain with two domains (Vκ-Cκ or Vλ-Cλ) [18]. IgM constant chain monomers show a greater degree of homology to IgEs than other isotypes. As shown in the alignments in Figure 3, the constant regions of the heavy chains, CH1, CH2, and CH3 of IgG correspond to the Cµ1, Cµ3, and Cµ4 of IgM. In contrast, the hinge region of IgG corresponds to the Cµ2 of IgM, which is an additional constant domain also found in other isotypes (mammalian IgE and avian IgY). It is thought that this domain functions much like the hinge region of IgGs and provides the flexibility needed to allow IgMs to bind multiple copies of antigens on cell surfaces. The heavy chains in each monomer are covalently linked with a disulfide bond at Cys 337 [19,71,72]. Each light chain is disulfide bonded to the heavy chain using cysteine residues at position 136 in the heavy chain [73].

Human immunoglobulin heavy chain constant domains are shown in Figure 3 below.

**Figure 4:**
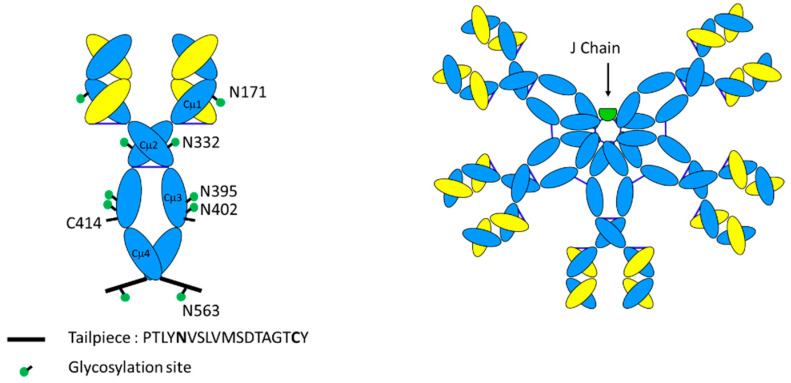
Schematic diagram of IgM monomer vs. IgM pentamer. IgM monomers are distinguished from IgG counterparts by their extensive glycosylation at the asparagine residues indicated, the presence of an additional domain Cµ2 in place of a hinge, and the presence of a short tailpiece peptide sequence that is critical for multimerization. Pentameric IgM has an additional 137 amino acid joining (J)-chain.

As shown in Figure 4, an additional feature of µ heavy chain is the presence of a short 18 amino acid peptide sequence (PTLYNVSLVMSDTAGTCY) at the C-terminus called the “tailpiece” [75]. IgM monomers are covalently linked by disulfide bonds between the penultimate cysteine of these tailpiece peptides. The tailpiece peptide is critical for IgM polymerization [76]. Indeed, the tailpiece can induce the polymerization when fused at the C-terminus of other antibody isotypes such as IgG [77]. In addition, inter-monomer disulfide bonds between Cys 414 residues in Cµ3 hold the center of the IgM in a well-defined ring-like structure.

**Figure 5:**
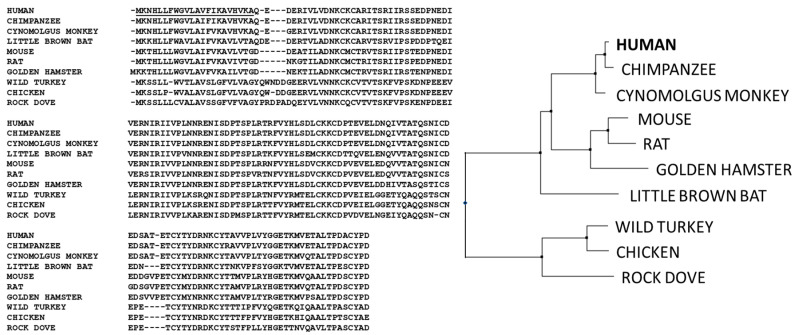
Sequence alignment (**left**) and hierarchical clustering (CLUSTALW, **right**) of IgM joining chains (J-chains) showing the high degree of conservation across species from human, primates, rodents, and birds.

In addition to the heavy chain and light chains, IgMs also possess a third chain, a polypeptide of 137 amino acids, known as the joining (J)-chain, which is a key feature of polymeric IgA and pentameric IgM antibodies [78,79]. The sequence of the J-chain is highly conserved from amphibians to humans [69], and is a distinct domain, unrelated to the immunoglobulin fold found in heavy chains and light chains (see Figure 5). There is a very high degree of sequence conservation within the J-chain, consistent with key structural and functional aspects of the J-chain integration into IgA and IgM oligomers [80,81] (see Section 3.3). The J-chain allows binding and transport of IgM pentamers and IgA oligomers to mucosal surfaces via interactions with polymeric Ig receptor (pIgR, see Section 3.3 and Section 4) [82].

### 3.2. Glycosylation

Antibodies are glycoproteins with N-linked glycosylation. In the case of IgG, there is N-linked glycosylation at Asn 297, which influences binding to Fc gamma receptors, and hence has a role in modulating antibody-dependent cell-based cytotoxicity (ADCC). Significantly, IgMs have more sites of glycosylation as compared with that of IgG. Whereas IgG heavy chains have a single glycosylation site, human and non-human primate IgM heavy chains exhibit five N-linked glycosylation sites at Asn 171 (Cµ1), Asn 332 (Cµ2), Asn 395, Asn 402 (both in Cµ3), and Asn 563, which is in the heavy chain tailpiece [83]. An additional glycosylation site is present on the J-chain at Asn 49. These glycans are considered to facilitate polymerization and assembly of the oligomeric IgM structure [84], as well as provide IgM with greater solubility and longer in vivo half-life [85]. There is no well characterized role for the glycosylation of IgMs in mediating effector function, as has been demonstrated for IgG. Recently, Colucci et al. showed a role for sialylation on IgM in mediating internalization on T cells and IgM mediated immune suppression [86].

The additional sites of glycosylation increase the complexity of IgM antibodies. Detailed site-specific carbohydrate analysis of IgM demonstrates that not all of the N-linked sites are similarly glycosylated. N-linked glycosylation has various levels of complexity, from high mannose or simple glycans to bi-, tri- and tetra-antennary complex glycans. Interestingly, the three sites at Asn 171, Asn 332, and Asn 395 (in domains 1, 2, and 3) exhibit complex carbohydrate moieties with sialylated termini. However, the more carboxy terminal sites at Asn 402 and Asn 563 (in domains 3 and 4) contain high mannose structures [84]. This pattern of glycosylation is consistent with the amino terminal regions of IgM being more accessible to glycosylation enzymes of the intracellular Golgi apparatus, whereas the carboxy terminal regions (the “central core” structure) are not fully processed, perhaps due to steric hindrance and lack of accessibility of these glycans in the oligomerized form of IgM. The fourth glycosylation site on IgM (Asn 402) is homologous to the single site of IgG (see Figure 3), which is known to have limited accessibility and does not exhibit fully developed complex carbohydrate in either IgM or IgG.

### 3.3. Tertiary Structure

The nearly mega dalton size of a fully assembled IgM complex has proven to be a challenge for determining a detailed structure. However, informative and useful models of IgM were initially created using a combination of techniques including low resolution cryo-electron microscopy, X-ray crystallography, NMR-derived structures of the subdomains, and homology modeling.

**Figure 6:**
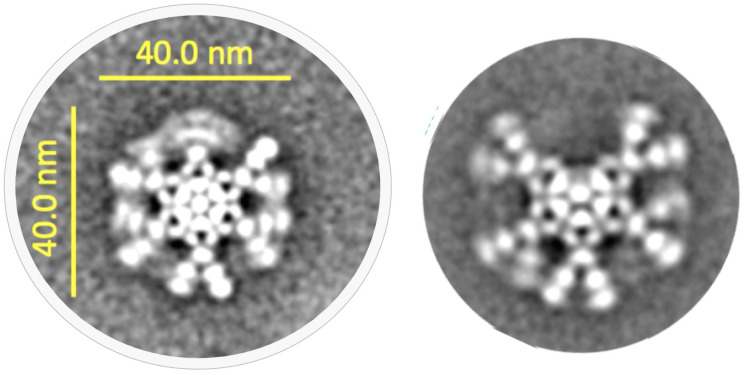
Example cryo-electron microscopy (cryo-EM) results for hexamer (**left**) and pentamer (**right**) forms of an anti-CD20 IgM. Consistent with published observations [91], the pentamer formed in the absence of J-chain retains the positions of monomers around the central ring and only a single monomer appears substituted by the J-chain. These images are a montage of a large series of negatively stained, transmission electron micrographs obtained via collaboration of IGM Biosciences and NanoImaging, Inc., San Diego, CA, USA (unpublished results).

In some of the earliest studies [87,88], three-dimensional (3D) models were proposed based on electron micrographs that placed the five monomers of a pentamer in a symmetrical structure around a central ring. Indeed, both planar (antigen-free) and “stable-like” (antigen-bound) structures for IgM antibodies were described. Subsequently, cryo-atomic force electron microscopy combined with the known crystal structure of IgE enabled further modeling of IgM structure [89]. Similar to the earlier models, these authors proposed a symmetrical distribution of five monomers around a central ring that exhibited a “flexural bias”, which allowed it to remain in a planar structure in the absence of antigen but, then, underwent a conformational change upon binding to antigen coated surfaces, such as those on cell or microbial surfaces. The “staple-like” structure that was formed then allowed binding of C1q, the first component of complement. Muller et al. assembled a model using crystal structure of domains Cμ1 and Cμ4 and an NMR-derived model of Cμ3 [90]. However, a recent study [91] with and without Fab arms attached to the central Fc ring, conclusively showed that the pentamer was actually an asymmetric structure where a single monomer from the hexamer was substituted with a J-chain without perturbing the position of the rest of the monomers (see Figure 6).

Interestingly, although the mu chain tail pieces and the J-chain are known to play critical roles in the assembly and function of IgM antibodies, for many years, the three-dimensional structures within the IgM pentamer have largely remained elusive. In fact, until recently, only a secondary structure for the J-chain has been proposed [92]. Importantly, in two new pivotal studies, the 3D structures of pentameric IgM [80] and human IgA [81] were reported. In these reports, Li et al. described the cryo-electron microscopy (cryo-EM) structure of an IgM Fc pentamer that included the J-chain and the ectodomain of pIgR, and Kumar et al. described the atomic structure of dimeric, tetrameric, and pentameric IgA Fc fragments linked by J-chain and in a complex with the secretory component of pIgR. These reports clearly show a unique “two-winged” structure for J-chain that is conserved and exhibits highly similar conformations within the IgM and IgA context. The J-chain binds to the Fcμ pentamer of IgM and forms clasp or bridge between the Fcμ1A and Fcμ5B monomers of the IgM via disulfide bonding of the tailpieces with J-chain cysteines [80]. With respect to the central structure of IgM, these models also show how the ten heavy chain tail pieces assemble within the central core of the Fc ring and interact with the J-chain. The tailpieces of IgM form parallel beta strands and the ten tailpieces pack in anti-parallel fashion. These authors suggested that the combined set of tailpieces formed prominent interactions that stabilized the pentamer while the J-chain served as a template for the oligomerization of IgM. These recent studies provide a fresh view of the structure of IgM antibodies and new functional insights into the unique biology of IgM and its interaction with the secretory pathway (see Section 4.3.1).

## 4. Function

### 4.1. Binding to Microbial Antigens, Role of Avidity

Natural IgM antibodies, in conjunction with natural killer (NK) cells, dendritic and mast cells, and macrophages are part of the innate immune system, the first line of defense against invading microorganisms and aberrant human cells (see Section 2.1 and Vollmers 2006 [93]). This response involves binding to specific antigenic motifs, such as specific carbohydrates on glycoproteins or glycolipids and repetitive structures such as lipopolysaccharides, recognized by IgM antibodies encoded by germ line (i.e., unmutated) genes. In so doing, these natural IgM antibodies play an important role in primary defense mechanisms, recognizing foreign bacteria and viruses or mutated human cells such as cancer cells. Typically, these natural IgM antibodies utilize low affinity binding to a range of similar foreign antigens, and their ability to eliminate these foreign antigens is, then, amplified by the high avidity afforded by having 10 (in the pentamer) or 12 (in the hexamer) binding sites. The potent ability of IgM antibodies to fix complement and opsonize particles make them particularly effective against bacteria and viruses [94]. The physical and functional characteristics of IgM and other antibody classes have been summarized by Strohl [95].

### 4.2. IgM vs. IgG Function: Complement Dependent Cytotoxicity (CDC) vs. Antibody-Dependent Cell-Based Cytotoxicity (ADCC)

**Figure 7:**
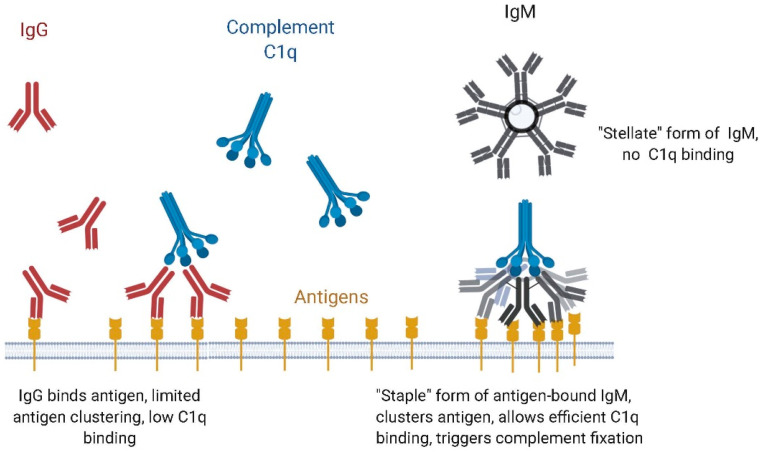
Complement binding and activation with IgM as compared with IgG.

IgM antibodies also differ from IgG isotypes due to the relative engagement of effector mechanisms. IgGs utilize natural killer cell engagement which can result in antibody-dependent cellular cytotoxicity (ADCC), as well as complement dependent cytotoxicity (CDC). In contrast, IgM does not bind the Fc gamma receptors, and therefore does not exhibit ADCC. However, IgMs have very potent CDC activity. Their hexameric or pentameric structure allows highly avid binding of complement component C1q to IgM, and therefore IgMs are able to fix complement substantially better than IgGs [96] (see Figure 7). Recent work by Sharp et al., using phase-plate cryo-electron microscopy, has provided a detailed model of how complement fixation was initiated with a large conformational change upon antigen binding, which exposed the regions on IgM that were bound by C1q, i.e., the first protein complex needed to initiate the complement cascade [97]. The planar or disc-like structure of free IgM changes to a “crouching” or “staple-like” structure when the Fab regions bind antigen on a cell surface. The antigen binding Fab regions move out of the plane of the ring formed by the Cµ3, Cµ4, and tailpiece due to the flexibility of the Cµ2 regions, which are the equivalent of the hinge regions of IgGs. This allows many or all of the Fab arms to contact antigens on a surface, leveraging the avidity of IgMs. Other effector mechanisms, such as antibody-dependent cellular phagocytosis, have also been implicated in the action of IgMs [98,99].

### 4.3. IgM Receptors: Structure and Tissue Distribution

**Figure 8:**
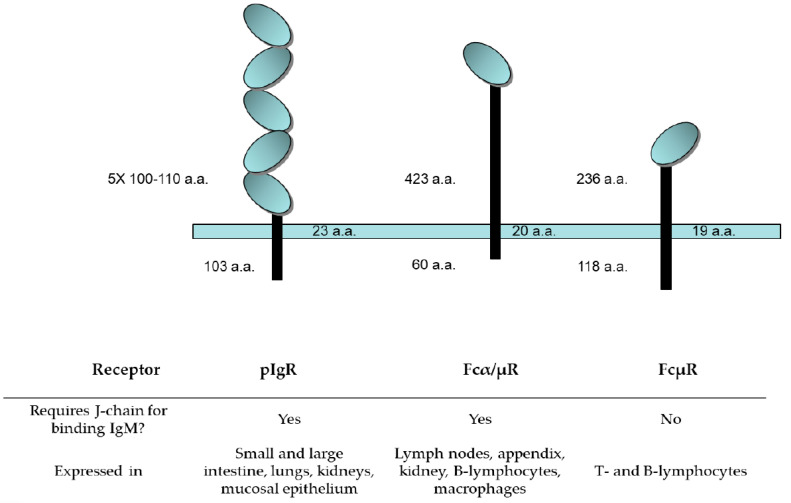
Schematic diagram of receptors known to bind IgM. IgMs bind at least three different receptors from those that bind IgG. The oval domains of each receptor indicate immunoglobulin fold-like regions. Their sizes and tissue distribution are depicted above.

IgM antibodies are known to bind to multiple receptors, which are illustrated in Figure 8. The functional roles of these three receptors are discussed below.

#### 4.3.1. Polymeric Ig Receptor (pIgR)

J-chain containing polymeric immunoglobulins such as IgA and IgM are often found on mucosal surfaces associated with a peptide called the secretory component (SC). The SC peptide is a proteolytic fragment of a cell surface receptor responsible for transport of polymeric Igs from the apical to mucosal surfaces [100]. The polymeric Ig receptor (pIgR) is expressed on the basolateral surfaces of mucosal epithelium, showing the highest expression in small and large intestines, with expression also seen in tissues such as lungs, pancreas, kidneys, and endometrium [101].

Structurally, pIgR belongs to the IgG superfamily with five Ig-like domains (D1–D5) that are heavily glycosylated (see Figure 8). Hinge regions are present between D1 and D2 and also between D3 and D4 [102]. Upon binding polymeric IgM or IgA antibodies containing J-chain, pIgR is internalized and transported by the endosome from the basal to the apical surface [103]. A membrane-proximal region contains a proteolytically sensitive site that is cleaved when the endosomes are trafficked to the apical side and this cleavage results in the release of polymeric Ig bound to the ectodomain of pIgR, which is known as the secretory component. Free SC can also be released at the apical side as an 8 kDa fragment.

The crystal structure of domain 1 (D1) of pIgR reveals a structural similarity to the variable domain of Ig that contains a highly conserved helix with a region that is implicated in binding to IgM [104]. The D1 of secretory component is both necessary and sufficient for IgM (or IgA) binding, however D2 through D5 contribute to increased affinity. The apoSC protein forms a compact structure in the absence of IgM or IgA but undergoes a drastic conformational change upon binding to polymeric IgM or IgA. The interaction of Fcμ of IgM with J-chain and pIgR form a ternary complex (Fcμ-J-SC) that facilitates transport of IgM (and IgA) to the apical side of epithelial cells. The molecular mechanism of pIgR/SC secretion of IgM (and IgA) is not fully understood. However, recent reports on 3D cryo-imaging of IgM with J-chain and pIgR/SC in complex, have contributed to a better understanding of both the structure and function of the secretory pathway components [80,81].

#### 4.3.2. Fcα/µR

The Fcα/µ receptor was identified in a screen of receptors from a cultivated mouse lymphoma cell line capable of binding IgMs. This receptor is approximately 70 kDa in size, belongs to the immunoglobulin superfamily, and is extensively glycosylated. The Fcα/µ receptor is localized to all lymphoid tissues including lymph nodes and the appendix, and is also widely expressed in non-lymphoid tissues including kidney and intestine, with lower expression observed in the lungs, liver, and myocardium [105]. Residues 76–98 are homologous to the CDR1 region of pIgR, which constitutes a conserved binding site for both proteins. The predominant cells expressing Fcα/µ receptors are the follicular dendritic cells in the germinal centers [106]. As with pIgR, the Fcα/µ receptor appears to interact with IgMs, primarily with determinants in Cµ3 and Cµ4 [107]. The presence of the Fcα/µ receptor on intestinal macrophages, plasma cells, and Paneth cells implicates its role in local and systemic aspects of mucosal immunity.

#### 4.3.3. FcµR, the TOSO Receptor

The most recently identified receptor interacting with IgM is FcµR, which is a transmembrane sialoglycoprotein of approximately 60 kDa [108]. FcµR, also known as the TOSO receptor, is highly expressed on chronic lymphocytic leukemia B cells and has been demonstrated to internalize upon IgM binding [109]. It is distinct from pIgR and Fcα/µ in that it only recognizes IgM and not polymeric IgA. The CDR1 region of FcμR that is predicted to recognize IgMs is very short, i.e., only five amino acids. Notably, FcµR does not require a J-chain for binding pIgM and its interactions are primarily thought to be with domains Cµ3 and µ4 [110]. Cells expressing FcµR were predominately adaptive immune cells, such as B and T cells [111].

## 5. Manufacturing Considerations

The need for scalable production processes will grow as the therapeutic interest in the use of IgM antibodies increases. IgMs have been considered to be difficult to express, due to their large size and complexity (see Table 1), resulting in low expression levels, and therefore expectations of a high cost of goods associated with therapeutic IgMs [112,113]. However, improvements in cell lines, production media, and process monitoring have made it such that production of a high-quality IgM is possible.

### 5.1. Expression of IgM

Biotherapeutic proteins, in general, including immunoglobulins, can be expressed in a variety of expression host cells [114]. Mammalian cells are typically used as host cells for IgM expression, in order to preserve the glycosylation patterns that are optimal for bioactivity or pharmacokinetic properties. Among mammalian cell hosts, Chinese hamster ovary (CHO) cells are most commonly used for producing antibodies because of the ability of these cells to grow in serum-free media at high density in large bioreactors [115]. CHO cell production of IgG antibodies has shown a steady improvement over the last 30 years of development and can reach a specific productivity of 50 to 60 picograms/cell/day and high titers of 10 to 15 g per liter. However, production of IgM antibodies in CHO cells is still a challenge. Kunert et al. first described the production of a class-switched anti-HIV IgM antibody, designated 4E10, in CHO-DUKX-B11 cells in serum containing medium, but were only able to achieve a specific productivity of 10 pg/cell/day [116]. To improve productivity and quality, Tchoudakova et al. utilized a different mammalian cell line, PER.C6, which was a transfected primary human embryonic retinoblast cell line, to make a panel of IgM antibodies and they were able to achieve a volumetric productivity of 20 pg/cell/day [117]. An additional host used for production of IgM is the tobacco plant [118]. By engineering in the expression of human sialyltransferase and galactosyltransferases, Loos et al. were able to demonstrate that fully functional IgMs with human-like glycoforms could be produced in tobacco plants.

The manufacture of IgM molecules for early clinical trials was done using hybridoma cells derived from rat or mouse myeloma cells or a heteromyeloma between human lymphoid cells and murine myeloma cells (see Table 3 in Section 6.1). Using these approaches, yields of 200 mg/L in batch process and 700 mg/L in a medium exchange process [117] were achieved. The two recombinantly expressed IgM antibodies, PAT-SC1 and PAT-SM6, were produced in PER.C6 cells and achieved fed-batch titers of 800 to 900 mg/L [117,119].

### 5.2. Purification of IgM

The purification of IgM for Good Manufacturing Practice (GMP) manufacturing has not been able to take advantage of affinity resins such as Protein A, which has been the standard recovery method for IgG. IgM does not bind to protein A. However, other affinity resins are available and are quite useful for research scale purification but are not currently available for scale-up with an associated GMP-compliant regulatory support file. IgMs also appear to have a narrower range of conditions under which they remain soluble as compared with IgGs, which can present difficulties in purification. Although low pH steps can be used for viral inactivation with IgMs, detergent-based approaches are more commonly used for viral inactivation in IgM downstream processing.

Early methods for purification of IgMs have included isoelectric precipitation and gel chromatography [120]. These investigators showed that product recovery of 40% could be achieved with 99% purity. For hybridoma cultures, polyethylene glycol (PEG) precipitation was optimized and combined with anion exchange chromatography for several antibodies. With the exception of two examples, greater than 95% purity with yields that varied from 28% to 84% was achieved using this approach [117]. This process was further improved by initially digesting the genomic DNA with the endonuclease benzonase.

In 2007, a three-step purification strategy for IgM antibody molecules was presented at a conference on purification of biological products [121]. The investigators used ceramic hydroxyapatite (CHT) chromatography for primary capture with a 90% purity and 79% recovery. The purification strategy subsequently used anion exchange (AEX) and cation exchange chromatography to achieve 99% purity with recovery of 50% to 80%. In 2010, Gagnon et al. reported the use of a monolithic anion exchanger with more than two-fold increased IgM dynamic binding capacity when compared to a porous particle anion exchange resin [122]. This approach was associated with greater genomic DNA removal due in part to the 15-fold higher charge density of the monolith exchanger. These results suggest that the convective nature of the monolithic matrix, rather than diffusion in porous resin, was perhaps better suited for IgM purification. This process was utilized at the 250 L scale with PER.C6 cell expression for producing PAT-SM6 which, at the time, was under evaluation in a Phase 1 melanoma study [119]. In this downstream process, CHT chromatography was used as the primary capture column with viral inactivation performed utilizing Triton X-100 wash step on column. Then, the DNA level was reduced with a Sartobind Q membrane, followed in succession by anion and cation exchange monolithic chromatography. The overall process yield was reported to be 55%.

Large scale GMP manufacture of IgM products is possible with a variety of traditional columns. New mixed-mode resins also may provide even greater capabilities. In 2011, at an IgM meeting in Germany, GE Healthcare reported on the use of layered beads in which the inner core was functionalized and the outer core was inert and porous [123]. This led to the launch of CaptoCore 700 and, more recently, CaptoCore 400 with an inert shell acting in a size exclusion mode and an anionic core, which are ideal for the large IgM molecule. Purification matrices such as these, along with bringing affinity resins to a scalable, regulatory-compliant state, should make the purification of clinical IgM antibodies more tractable with high yields and good safety clearance.

## 6. Therapeutic Uses of IgM Antibodies

**Figure 9:**
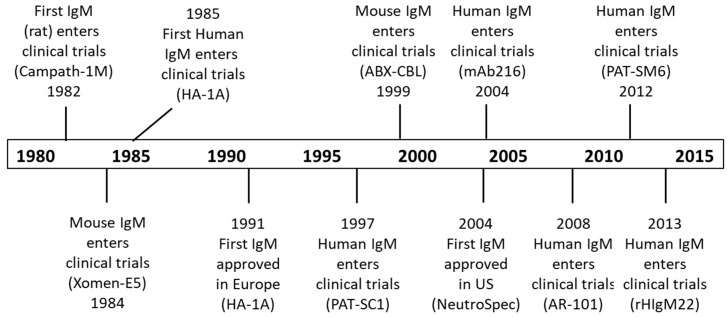
History of selected IgM human clinical trials.

As has been well demonstrated with IgG antibodies, IgM antibodies also have the potential to provide therapeutic benefit in humans. Indeed, IgM antibodies have been shown to be efficacious in a variety of animal models, including non-human primates [124] and were some of the first mAbs to be tested clinically (see Figure 9 and [125]).

Currently, there are hundreds of therapeutic IgG antibodies that have advanced to clinical trials, and more than 90 antibody-based products have achieved FDA approval [126]. However, only about 20 IgM antibodies have been tested in humans (Table 2). Included in this group are rat, mouse, and human IgMs that target a variety of infectious disease, oncology, and autoimmune disease antigens.

Taken together, the studies described below demonstrate that IgM antibodies can be safely administered to humans. However, the IgM antibodies tested to date have not typically produced sufficient efficacy in humans to obtain (or maintain) regulatory approval. This result is likely due to the fact that most, if not all, of the IgM antibodies tested were of natural origin and, as a consequence, essentially contained germ-line gene sequences that have not undergone extensive somatic mutation, and thus were of low affinity and specificity [127]. It is also likely that the particular indications tested in these early studies, such as a major focus on sepsis and septic shock, has limited the ability of IgM antibodies to achieve regulatory success.

### 6.1. IgM Clinical Trials

Shown in Table 3 are additional details regarding the IgM mAbs so far examined in humans, organized by the nature of the target antigens. For all of these studies, administration of the IgM antibodies was well tolerated. Of particular interest is the fact that more than half of these IgMs target antigens that are poorly immunogenic and for which it has been difficult to generate IgG mAbs [128]. Included in this category are lipopolysaccharides (LPS) and its component core structure lipid A, gangliosides, proteolipids, and glycans. Since many of these structures are composed of polymeric or repeated antigenic motifs, the avidity effects of having 10 binding sites on the IgM antibody may well provide significant advantages for such antigens over their IgG counterparts. It is apparent, however, that the approaches for finding such antibodies need to be changed significantly, focusing on the incorporation of affinity optimized V domains into the IgM backbone rather than using naturally occurring IgMs.

#### 6.1.1. Lipopolysaccharide Antigens

Five of the IgM product candidates (from a total of nine mAbs) in Table 3 targeted lipopolysaccharide (LPS), a component of the Gram-negative bacterial cell outer membrane, and two of these antibodies, E5 and HA-1A, were some of the earliest and most extensively studied IgM antibodies to enter clinical trials. LPS is highly inflammatory and has been the subject of numerous interventional strategies. E5, a murine anti-lipid A IgM mAb isolated by Lowell Young at UCLA (U.S. patent 4918163) and licensed to Xoma (Xomen-E5), and HA-1A, a human anti-lipid A IgM mAb isolated by Nelson Teng at Stanford [146] and licensed to Centocor (as Centoxin), entered clinical trials for sepsis in the early 1980s; both IgMs were evaluated in a number of clinical trials, and product license applications (PLAs) for both products were submitted to the FDA in early 1989 [147]. Centoxin^TM^ (nebacumab) received regulatory approval in Europe in 1992. However, this approval was withdrawn in 1993, following the inability of subsequent trials to demonstrate a clinical benefit [147].

Around this same time, several additional clinical trials were initiated with other anti-LPS antibodies. Chiron initiated a Phase 1 trial with MAB-T88, and reported that it was safe and well tolerated [131]. A second Phase 1 study was also conducted in six sepsis patients with a high likelihood of Gram-negative bacteremia [148]. MAB-T88 was again shown to be safe, but additional clinical trials were never conducted. Similarly, a cocktail of five human IgM anti-*Pseudomonas aeruginosa* LPS antibodies was tested in normal adults and in patients with *P. aeruginosa* bacteremia [132]. This cocktail also appears to have been well tolerated, but additional studies do not appear to have been conducted. More recently, Aridis tested AR-101, an anti-*P. aeruginosa* LPS IgM originally developed by Kenta (KBPA-101, panobacumab) [149], in patients with nosocomial pneumonia. These studies went as far as a Phase 2a trial [133], but more recent efforts appear to have focused on the IgG anti-LPS mAb AR-105 [150]. Similarly, multiple clinical trials have also been completed [151] or are in progress [152], using IgM-enriched IVIG for the treatment of sepsis or septic shock.

While the above results are, at first, discouraging, it is also now clear that many of the issues associated with the anti-LPS IgM mAb trials likely reflect the difficult nature of this clinical indication (numerous other therapeutics have failed in sepsis and infectious disease trials) [153] as well as the specific characteristics of the natural, non-affinity-matured mAbs tested [124].

#### 6.1.2. Glycolipid and Proteolipid Antigens

Another three IgMs, in Table 3, target gangliosides or proteolipids. Of the two IgMs targeting gangliosides, L612 targets ganglioside GM3, while MORAb-028 targets ganglioside GD2. Antibody L612 was derived from Epstein–Bar virus (EBV)-transformed B cells from a patient with melanoma, and was shown to kill melanoma cells via complement-dependent cytotoxicity (CDC) [154]. However, when tested clinically in patients with melanoma, L612 showed no adverse side effects but lacked evidence of efficacy [134]. Subsequently, and because it lacked a J-chain, L612 preparations were found to contain roughly 20% hexameric and 74% pentameric forms of the IgM [155]. Since the hexameric form of L612 appeared to exhibit most of the CDC activity, a recombinant hexamer-dominant form of L612, CA19, was selected and produced approximately 80% hexamer from CHO cells. While promising in animals, CA19 does not appear to have been tested in the clinic.

MORAb-028 is an IgM that targets ganglioside GD2 licensed from Micromet and originally designated MT228. MORAb-028 entered two Phase 1 clinical trials in 2010, one for intratumoral injection [135] and one for IV administration of radiolabeled MORAb-028 [156]. The studies were both completed in 2012, but little information regarding their results is available. The program appears to have been discontinued in 2014 [157].

rHIgM22 binds to a complex myelin proteolipid antigen that is only expressed in CNS white matter and has been reported to promote remyelination in animal models [158]. It was developed at the Mayo Clinic and licensed to Acorda. rHIgM22 has been investigated following IV infusion in two Phase 1 studies in patients with multiple sclerosis, one starting in 2013 [159] and one in 2015 [160]. The results for the first study have been published [136]. In this study, rHIgM22 was well tolerated in the 55 patients treated, and the IgM was detected in the CSF, but no statistically significant changes were observed in the exploratory outcome measures.

#### 6.1.3. Glycan Antigens

Despite the fact that IgM antibodies are well suited to target repetitive antigens [161], very few clinical trials testing carbohydrate-reactive IgM mAbs have been conducted to date. Two such studies are listed in Table 3. MAb216 recognizes a blood group antigen (CDIM) that is present on human B cells [162]. This antibody, also obtained by Nelsen Teng and colleagues at Stanford, was isolated from a patient with lymphoma and, after scale up at the NCI, was tested in a small Phase I clinical trial in patients with B cell acute lymphoblastic leukemia. While the results were encouraging [139], limited production of mAb216 by heteromyeloma cells inhibited further testing.

On the basis of these results, a recombinant human IgM variant of mAb216, termed IGM-55.5, was generated. The antigen recognized by both mAb216 and IGM-55.5 on human B cells is a linear lactosamine epitope that is sensitive to the enzyme endo-beta-galactosidase. This ligand, termed “cell death inducing molecule” (CDIM), is similar to the “i” antigen of cord blood red blood cells [163,164]. The “i” antigen is only found on the red blood cells of the developing fetus and newborn infants and, in rare cases, in human adults whose red blood cells did not convert this simple linear carbohydrate into the more complex branched carbohydrate named “I” antigen. Natural autoantibodies to the “i-antigen” often circulate in the blood of healthy adults and are usually of the IgM isotype.

Interestingly, IgM antibodies of this class often have heavy chain variable regions encoded by the human Vh4-34 V region gene [165], and they are able to kill antigen-expressing B cells via the formation of large complement-independent pores [166]. This process, which has also been reported for other IgM antibodies [167,168] and has similarities with oncosis, involves ”wounding” target cells in a complement-independent manner such that large holes or pores are formed in the cell membrane. The precise mechanism by which these IgM antibodies mediate cell killing is not yet known, but it has been speculated that degradation of actin-associated proteins permits the aggregation of membrane components, thus leading to the formation of pores and loss of intracellular contents [168].

Patrys Ltd. in Australia was one of the first companies to focus on investigating the therapeutic potential of natural human IgM antibodies, and two of the candidates tested clinical target glycan-based epitopes. PAT-SC1, originally isolated by Peter Vollmers and colleagues at the Institute of Pathology at the University of Würzburg [169] targeted a specific glycoform on CD55 that appeared to be overexpressed on the surface of many cancer cells. PAT-SM6, also isolated by Vollmers [170], targeted an O-linked glycoform on GRP78, a multifunctional glucose-regulated protein that was possibly only present on tumor cells. Both PAT-SC1 [138] and PAT-SM6 [140] have completed Phase 1 trials, and both appear to have been well tolerated. Currently, only PAT-SC1 is still under development, having been licensed to Hefei Co-source Biomedical Co. in 2015, for all oncology indications in China [171].

Lastly, NeutroSpec^TM^ (fanolesomab-Tc99m) is a radioimmunodiagnostic agent consisting of a murine IgM monoclonal antibody labeled with technetium-99m (^99m^Tc). Fanolesomab is directed against the carbohydrate moiety 3-fucosyl-N-acetyl lactosamine that defines the cluster of differentiation 15 (CD15) antigen (NeutroSpec^TM^ package insert) [172]. The CD15 antigen is expressed on the surface of polymorphonuclear neutrophils (PMNs), eosinophils, and monocytes, cells that are often localized in sites of infection. Initial clinical trials have indicated product safety [137] and, in 2004, NeutroSpec^TM^ received FDA approval for scintigraphic imaging of patients with equivocal signs and symptoms of appendicitis who were five years of age or older. However, the product was suspended in 2005 following reports that patients taking the drug suffered serious and life-threatening cardiopulmonary events. NeutroSpec^TM^ was subsequently discontinued in 2008.

#### 6.1.4. Protein Antigens

One of the first IgM antibodies to be tested clinically was Campath-1M. This antibody, which recognized the lymphocyte antigen CD52, was an IgM mAb isolated by Herman Waldmann and colleagues from rats immunized with human lymphocytes [125]. Early clinical trials for the prevention of graft vs. host disease (GvHD) involved the ex vivo purging of donor allographs with Campath-1M plus complement were encouraging, and two patients (one with non-Hodgkin’s lymphoma and one with acute lymphoblastic leukemia) received intravenous infusions with Campath-1M [125]. However, overall efficacy of the treatments was low and there were concerns regarding immunogenicity of the rat IgM [173].

Ultimately, Campath-1M was first class-switched to a rat IgG2b (Campath-1G) [174], and then became the first antibody to be humanized by successful transplantation of the six heavy and light chain variable regions from the rat IgG2b mAb into a human IgG1 [175], creating Campath-1H. Campath-1H was subsequently shown to be safe and effective in humans and is currently marketed under the trade name Lemtrada^®^ (alemtuzumab) for B cell chronic lymphocytic leukemia [176].

In addition to Campath, four other IgM antibodies to protein antigen targets have been tested in clinical trials. Two of these IgM mAbs are of mouse origin (ABX-CBL, TOL101), one is chimeric (ARG098) and one is human (Mab 16.88). ABX-CBL (murine hybridoma-derived IgM) and TOL101 target human CD147 and the αβ T cell receptor, respectively. ABX-CBL was tested in patients with steroid-refractory acute graft-versus-host disease (aGvHD) at doses up to 0.3 mg/kg/day [143]. Among 51 evaluable patients in the Phase 1 study, roughly half (51%) responded following nine daily doses. However, in a randomized Phase 2/3 clinical trial (95 patients) in acute GvHD comparing ABX-CBL to standard of care, anti-thymocyte globulin (ATG), the patient outcomes were insignificantly different [177]. These data indicated that ABX-CBL did not offer improvement over ATG and as a result, further clinical development of ABX-CBL was terminated.

TOL101 targets the human αβ T cell receptor and was tested in renal transplant patients. Interestingly, in an effort to minimize T cell activation and its consequences that were observed with higher affinity IgG antibodies, this IgM was explored as a lower affinity/lower avidity therapeutic targeting this antigen. In a Phase 2 study [178], patients received five daily doses, up to 42 mg/day, and prolonged CD3 modulation occurred at doses above 28 mg. There were no cases of patient or graft loss, the treatments were well tolerated, and CD3 levels recovered within seven days after the cessation of therapy. No additional updates were found.

ARG098 is a mouse/human chimeric IgM antibody that targets FAS receptor and was tested in subjects with rheumatoid arthritis. Unlike the other IgM antibodies discussed here, ARG098 was administered via intraarticular injection into the knee at very low doses (up to 10 µg per knee). As ARG098 exhibited evidence of clinical activity, a placebo-controlled Phase 2a study was initiated and the program was partnered with Centocor, however trials were apparently discontinued in 2015 [179].

Lastly, 16.88 is a human IgM antibody that was derived from colorectal cancer patients immunized with autologous tumor cells admixed with BCG [180]. Of relevance to the current discussion is that all 13 of the natural human antibodies isolated in these studies, including 16.88, were of the IgM isotype. Following several pharmacokinetic studies in mice and humans [181], considerable efforts were made to examine the pharmacokinetics and tissue distribution of radiolabeled 16.88 in humans [182]. These studies demonstrated that 16.88 effectively targeted human tumors, and that it may be useful for radioimmunotherapy, but such studies were apparently not conducted.

### 6.2. IgM Pharmacokinetics

In 1964, Barth and colleagues published one of the first articles examining the pharmacokinetics (PK) of normal, unaltered human IgM antibodies in humans [183]. The IgM test material was purified from the serum of a healthy donor, radiolabeled with iodine-131, and then injected into seven normal adults. Serum samples were collected daily and analyzed in a gamma counter. According to these studies, the terminal half-life of normal human IgM in humans was calculated to be 5.1 days, with a range of 3.8 to 6.5 days (Table 4). Notably, these values for IgM half-life are four-fold less than the half-lives commonly reported for human IgGs in humans (e.g., 18–21 days) [184], most likely reflecting the fact that IgM antibodies do not bind to the recycling FcRn receptor (see Section 4.2).

The pharmacokinetics of several therapeutic IgM mAbs have also been studied in some of the clinical trials described in Section 6.1 (see Table 3). In general, the half-lives reported for these IgMs in humans are shorter than that described for the preparation of normal human IgM tested previously (Table 4). Importantly, it should be noted that there are several critical differences between the IgM antibodies tested clinically and the prior preparation used for human PK studies. First, the material tested by Barth was pooled normal human IgM and, as such, it would not bind to human antigens, whereas many of the other IgMs subsequently tested were selected for binding to human antigens. As a consequence, the clinically tested IgMs would bind to tissues expressing those targets and would likely be cleared more quickly. Second, the material tested by Barth was isolated from human serum, whereas most of the other IgMs were produced in mouse, rat, or hamster (e.g., CHO) cells. Since changes in production host cells and culture conditions for IgGs are known to result in changes in glycosylation [188], and similar changes have been noted with IgM antibodies [189], such differences in PK are not unanticipated. Lastly, differences in analytical techniques (isotope vs. ELISA) and subject populations (normal vs. diseased) are also contributing factors.

Combined, these differences make direct comparisons of the reported data quite difficult, not only between trials but also with the published data for normal human IgM. However, despite these differences it is encouraging to note that IgM antibodies can have relatively long half-lives in humans, thereby allowing weekly or bi-weekly dosing in the clinic.

### 6.3. IgM Safety and Immunogenicity

As indicated in Table 3, a number of clinical trials have been conducted with rodent or human IgM antibodies in a range of clinical indications. For these trials, nearly 400 subjects were treated with doses up to 27 mg/kg, and no apparent safety issues were reported. Importantly, for the studies conducted with human IgM antibodies, little or no immune responses were noted. However, it should be emphasized that the specifics of the immunogenicity assays used, as well as their relative sensitivities, were not typically reported.

Of the IgM antibodies listed in Table 3, two products (E5 and HA-1A) were tested in Phase 2 and Phase 3 clinical trials that enrolled a large number of patients. Both of these antibodies target LPS, the outer-most layer on Gram-negative bacteria, and were tested in sepsis patients. In the Phase 3 trials alone, E5 was administered to approximately 715 patients [190,191], and HA-1A was administered to approximately 730 patients [192,193]. Thus, when combined with the subjects listed in Table 3 (*n* = 398), the total number of subjects treated with IgM antibodies was more than 1800 patients.

The observations that several human IgM antibodies have been safely administered in the clinic are particularly encouraging, given the theoretical concern that multivalent, high-avidity antibodies may exhibit off-target binding that could result in unexpected toxicities or rapid clearance. In some of the IgMs isolated as naturally occurring antibodies to tumor targets, there may be low affinity binding with high avidity which may contribute to unexpected, off-target binding. In the clinical studies reported to date, no such concerns have been raised. However, these concerns can only be addressed by further development and clinical testing of additional IgM antibody product candidates.

### 6.4. Other Oligomeric Antibody Forms

In addition to the more traditional IgM antibodies, a number of new molecular constructs have been generated that seek to approximate the hexameric structure of the IgM molecule. One such class of molecules, the HexaBody™, was generated by introducing mutations in the IgG heavy chain that allow oligomers up to hexamers form in a concentration-dependent fashion on the surface of cells [194]. The most advanced HexaBody™ in development is GEN1029, a mixture of two noncompeting anti-DR5 HexaBody™ molecules. A Phase 1/2 study of GEN1029 in patients with solid cancers was initiated in May, 2018 [195].

## 7. Future Applications of Therapeutic IgM

As our understanding of expression systems and manufacturing of IgM antibodies progresses, we anticipate the utilization of IgM as a new modality of engineered antibodies for treatment of various therapeutic indications. Most importantly, IgM has 10 or 12 binding sites and is capable of binding its antigen targets with high avidity. For cell surface targets where there is repetitive display on a cancer or other target cell, high avidity allows for multiple antigen engagements per IgM. As a consequence, IgMs are particularly well suited for targeting difficult antigens. In some earlier IgM-based development efforts (Section 6.1.1 and Section 6.1.2), antibodies against tumor antigens consisting of carbohydrate moieties or glycolipids were evaluated in clinical trials. In these cases, the affinity of corresponding IgGs on these glycotopes can be insufficient for effective targeting, whereas the IgMs exhibit strong binding and effector function appropriate for biotherapeutic use. Another challenging aspect of selected-tumor targets is often the low expression observed on tumors, especially treatment-resistant tumors. IgM-based antitumor agents with high avidity may yield antibodies with increased potency on low expression or otherwise difficult targets.

Given the greater valency of IgM, these macromolecules offer considerable opportunity for higher order cross-linking of cell surface receptors. In addition, the flexibility of the IgM may provide the appropriate architecture for binding multiple targets on a cell surface. The potential for IgM-induced multimerization of cell surface targets makes the IgM an ideal candidate platform for developing TNF receptor superfamily agonists. For example, IgM antibodies directed to death receptor 4 [196] have shown excellent efficacy in vitro and in vivo. Wang et al. also demonstrated significant potency and enhanced efficacy with IgMs specific for death receptor 5 as compared with the corresponding agonist IgGs [197]. In subsequent investigations, Wang, et al. demonstrated strong in vivo efficacy on established tumors that exhibited resistance to anti-DR5 IgG therapy in murine xenograft models [198]. Similarly, recent studies with IgM antibodies targeting the receptor binding site of influenza B have shown excellent potency and broad cross-reactivity in vitro and in animal models [199].

Many of the earlier programs that tested IgM in human clinical trials used natural IgM antibodies often isolated from patients or humanized from a murine hybridoma. However, there is significant opportunity for more engineered versions of IgM, where the variable domains of an affinity matured IgG can be grafted onto IgM constant domains. This “domain swap” of affinity matured variable domains from IgG onto the backbone of IgM can lead to marked increases in binding avidity and potency of an engineered IgM. As a platform for engineering oligomeric binding units, IgM offers a much wider variety of multimeric interaction with antigens.

Although engineering of antigen binding sites can yield novel IgM constructs with improved antigen binding, there also exists additional unique sites on IgM for adding multispecific binding. For example, bispecific IgG antibodies and other bispecific variants of IgG exhibit extremely potent tumor targeting agents [200]. However, these antibodies have just a single binding site to a tumor antigen, instead of the two binding sites of a traditional IgG. In contrast, a bispecific IgM may allow very high avidity binding to difficult or rare tumor antigens, with selective engagement of T cells for efficient tumor cell killing. We found that fusion of a single CD3 binding domain to the J-chain allowed for the production of engineered bispecific IgM antibodies that exhibited controlled engagement of T cells. For example, we recently described the use of a CD3 binding unit fused to the J-chain to generate T cell-engaging bispecific IgM antibodies that contained 10 binding sites for a cancer antigen and a single binding site for CD3 [201]. A key feature of this approach was the ability to make fully assembled bispecific IgM antibodies in a single, high expressing cell line.

One such antibody, IGM-2323, is an anti-CD20 x CD3 IgM with “10 × 1” bispecificity (10 binding sites for CD20 and one binding site for CD3ε) [202]. This novel bispecific IgM has very potent activity via T-cell directed cytotoxicity (TDCC), and it also retains the robust CDC activity typical of an IgM. Importantly, this IgM platform for T cell engagement exhibits potent TDCC via a mechanism that does not lead to high levels of cytokine release in vitro or in animals. On the basis of these properties, IGM-2323 is currently being tested in clinical trials for treatment of refractory or resistant non-Hodgkin’s lymphoma [202].

With renewed focus on IgM antibodies and the engineering of IgM antibodies, there may well be advantages inherent to the IgM platform that can yield improved biotherapeutic agents for treatment of unmet medical needs. The recently published three-dimensional structure of IgM Fc pentamer may also allow better understanding of this complex macromolecule [80]. We anticipate that the higher order valency of IgM with enhanced receptor cross-linking and the highly effective bispecific IgMs should provide new opportunities for antibody engineering and the development of more effective therapeutics.

## Figures and Tables

**Table 1:** IgM antibody complexity.

IgM Form	Molecular Weight	Peptides in IgM Complex	Inter-Chain Disulfide Bonds	N-Linked Sites of Glycosylation
Pentamer (with J-chain)	950 kD	21	27	51
Hexamer (without J-chain)	1150 kD	24	30	60

**Table 2:** Overview of IgM antibodies tested in human clinical trials.

Antibody (Name)	Company	IgM Source	Antigen	Indication	Most Advanced Clinical Development
Campath-1M	Academic (MRC-RDCT)	Rat	CD52	Graft vs. host disease	Phase 2
E5 (Xomen-E5)	XOMA	Mouse	J5 lipid A	Sepsis	Phase 3
HA-1A (Centoxin)	Centocor	Human	J5 lipid A	Sepsis	Phase 3
Fanolesomab-Tc99 (NeutroSpec)	Palatin	Mouse	CD15	Appendicitis	Phase 3
IgM cocktail (5)	Cutter/Miles	Human	LPS	Sepsis	Phase 1
Mab 16.88	Academic (Free University Hospital)	Human	Colon cancer antigen	Colorectal cancer	Phase 1
MAB-T88	Chiron	Human	LPS	Neutropenia	Phase 1
PAT-SC1	Patrys	Human	CD55 isoform	Gastric cancer	Phase 1
ABX-CBL	Abgenix	Mouse	CD147	Graft vs. host disease	Phase 2/3
L612	Chugai	Human	Ganglioside GM3	Melanoma	Phase 1
MORAb-028	Morphotek/Eisai	Human	Ganglioside GD2	Melanoma	Phase 1
AR-101	Aridis	Human	LPS	Nosocomial P. a. pneumonia	Phase 2a
mAb216	Academic (Stanford)	Human	CDIM	B-lineage ALL	Phase 1
PAT-SM6	Patrys	Human	GRP78	Multiple myeloma	Phase 1/2a
ARG098	Argenes	Mouse/Human (chimeric)	FAS	Rheumatoid arthritis	Phase 1/2
rHIgM22	Acorda	Human	CNS myelin	Multiple sclerosis/neuronal degeneration	Phase 1
TOL101	Tolera	Mouse	αβ TCR	Renal transplant	Phase 2

**Table 3:** Source, production and immunogenicity of IgM antibodies in early phase clinical trials.

Target Antigen Class	Antibody	Antigen	IgM Source	Production Cell	Indication	Clinical Trial	Dose	Immunogenicity in Humans	Reference
**Lipopolysaccharide**	E5	J5 lipid A	Mouse B cells	Hybridoma with mouse myeloma	Sepsis	Phase 1	0.1, 0.5, 2, 7.5, 15 mg/kg	3 of 9 subjects	Harkonen 1988 [129]
	HA-1A	J5 lipid A	Human B cells	Heteromyeloma with lymphoma spleen cells	Sepsis	Phase 1	25, 100, 250 mg	0 of 34 subjects	Fisher 1990 [130]
	MAB-T88	LPS	Human B cells	Hybridoma with mouse myeloma	Neutropenia	Phase 1	1, 4, 8 mg/kg single dose	0 of 9 subjects	Daifuku 1992 [131]
	Mab cocktail (5 IgM)	LPS	Human B cells		Normal adults P. aeruginosa bacteremia	Phase 1	0.75 to 3.0 mg/kg	12 subjects 8 subjects	Saravolatz 1991 [132]
	AR-101 (KBPA-101)	LPS	Human B cells	Hybridoma with mouse myeloma	Nosocomial P. aeruginosa pneumonia	Phase 2a	1.2 mg/kg × 3	0 of 18 subjects	Lu 2011 [133]
**Glycolipid/** **Proteolipid**	L612	Ganglioside GM3	Human B cells	EBV-transformed patient B cells	Melanoma	Phase 1	960, 1440, 1920 mg 48 h infusion	0 of 9 subjects	Irie 2004 [134]
	MORAb-028	Ganglioside GD2	Human B cells	Hybridoma with human/mouse myeloma	Melanoma	Phase 1	1 or 2 mg/cm^2^/day × 5 days, repeated 2×	18 subjects	NCT-01123304 [135]
	rHIgM22	CNS myelin proteolipid	Human B cells	Hybridoma with mouse myeloma	Multiple sclerosis/neuronal degeneration diseases	Phase 1	0.025 to 2 mg/kgsingle dose	55 subjects	Eisen 2017 [136]
**Glycan**	Fanolesomab-Tc99	CD15 (carbohydrate)	Mouse B cells	Hybridoma with mouse myeloma	Healthy volunteers	Phase 1	125 µg × 2 (21 days apart)	5 of 30 subjects	Line 2004 [137]
	PAT-SC1	CD55 (glycan isoform)	Human B cells	Recombinant production Per.C6 cells	Gastric cancer	Phase 1	20 mg single dose	51 subjects	Hensel 2014 [138]
	mAb216	CDIM (carbohydrate)	Human B cells	Heteromyeloma with lymphoma spleen cells	B-lineage ALL	Phase 1	1.25 mg/kg to 5 mg/kg 3 + 2 dose escalation	0 of 13 subjects	Liedtke 2012 [139]
	PAT-SM6	GRP78 (O-linked glycan)	Human B cells	Recombinant production Per.C6 cells	Multiple myeloma	Phase 1	0.3, 1 3 or 6 mg/kg 4 doses over 2 weeks	0 of 12 subjects	Rasche 2015 [140]
**Protein**	Campath-1M	CD52	Rat B cells	Hybridoma with rat myeloma	Graft vs. host disease	Phase 2	25 mg bid × 10	(not tested)	Friend 1989 [141]
	Mab 16.88	Colon cancer antigen	Human B cells	Hybridoma with mouse myeloma	Colorectal cancer	Phase 1	8 mg, then 200, 500 or 1000 mg	0 of 20 subjects	Haisma 1991 [142]
	ABX-CBL	CD147	Mouse B cells	Hybridoma with mouse myeloma	Graft vs. host disease	Phase 1	0.2 to 0.3 mg/kg 9 doses	0 of 51 subjects	Deeg 2001 [143]
	TOL101	αβ TCR	Mouse B cells	Hybridoma with mouse myeloma	Renal transplant	Phase 2	0.3, 1.4, 7, 14, 28, 42 mg 5 daily doses	1 of 36 subjects	Getts 2014 [144]
	ARG098	FAS	Mouse: Human B cells (chimeric)	Hybridoma with mouse myeloma	Rheumatoid arthritis	Phase 1/2	up to 10 μg/knee (intraarticular)	43 subjects	Matsubara 2013 [145]

**Table 4:** Pharmacokinetics of IgM antibodies in humans.

Antibody	Antigen	Indication	Model	Terminal Half-Life	Reference
Serum IgM (hu) I^131^-labeled	-	Humans	Two-compartment	5.1 days (122 h)	Barth 1964 [183]
E5 (mu)	LPS (Lipid A)	Sepsis	One-compartment	19.3 h	Harkonen 1988 [129]
HA-1A (hu)	LPS (Lipid A)	Sepsis	One-compartment	15.9 h	Fisher 1990 [130]
		Sepsis	One-compartment	14.5 h	Romano 1993 [185]
MAB-T88	Lipopolysaccharide	neutropenia	Two-compartment	41.5 h	Daifuku 1992 [131]
AR-101	Lipopolysaccharide	Nosocomial pneumonia	Two-compartment	102 h (after 3rd dose)	Lu 2011 [133]
5G2	LPS (O-side chain)	Sepsis	One-compartment	56 h	Meng 1993 [186]
rHIgM22	CNS myelin proteolipid	Multiple sclerosis	(not stated)	99 h (2 mg/kg)	Eisen 2017 [136]
ABX-CBL	CD147	GvHD	Two-compartment	15–19 h	Deeg 2001 [143]
TOL101	ab TCR	Renal transplant	One-compartment	23.8 h	Getts 2014 [144]
PAT-SM6	GRP-78	Multiple myeloma	(not stated)	5.9 to 8.4 h	Rasche 2015 [140]
Fanolesomab-Tc99	CD15	Healthy volunteers	Two-compartment	8 h	Package insert [187]
Mab 16.88	Colon cancer antigen	Cancer	(not stated)	20 h	Haisma 1990 [181]
